# Supplementary material for: Chemerin in Participants with or without Insulin Resistance and Diabetes
Source: Biomedicines. 2024 Apr 22;12(4):924. doi: 10.3390/biomedicines12040924 (PMC11048116; doi:10.3390/biomedicines12040924)
Supplement: Supplementary file 1 [file biomedicines-12-00924-s001.zip › biomedicines-2872615-supplementary.pptx]

## Slide 1
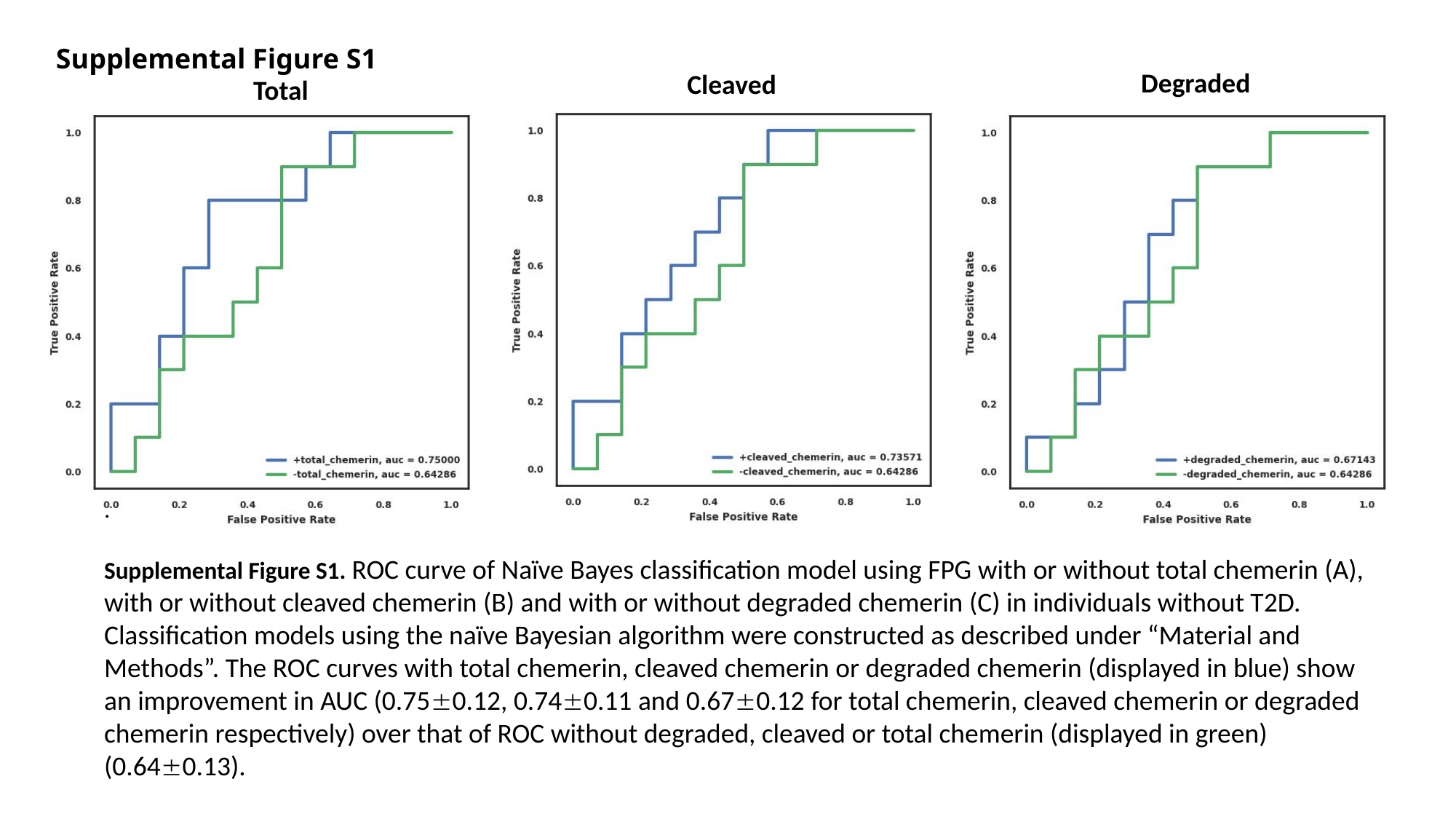

Supplemental Figure S1
Degraded
Cleaved
Total
.
Supplemental Figure S1. ROC curve of Naïve Bayes classification model using FPG with or without total chemerin (A), with or without cleaved chemerin (B) and with or without degraded chemerin (C) in individuals without T2D. Classification models using the naïve Bayesian algorithm were constructed as described under “Material and Methods”. The ROC curves with total chemerin, cleaved chemerin or degraded chemerin (displayed in blue) show an improvement in AUC (0.75±0.12, 0.74±0.11 and 0.67±0.12 for total chemerin, cleaved chemerin or degraded chemerin respectively) over that of ROC without degraded, cleaved or total chemerin (displayed in green) (0.64±0.13).

## Slide 2
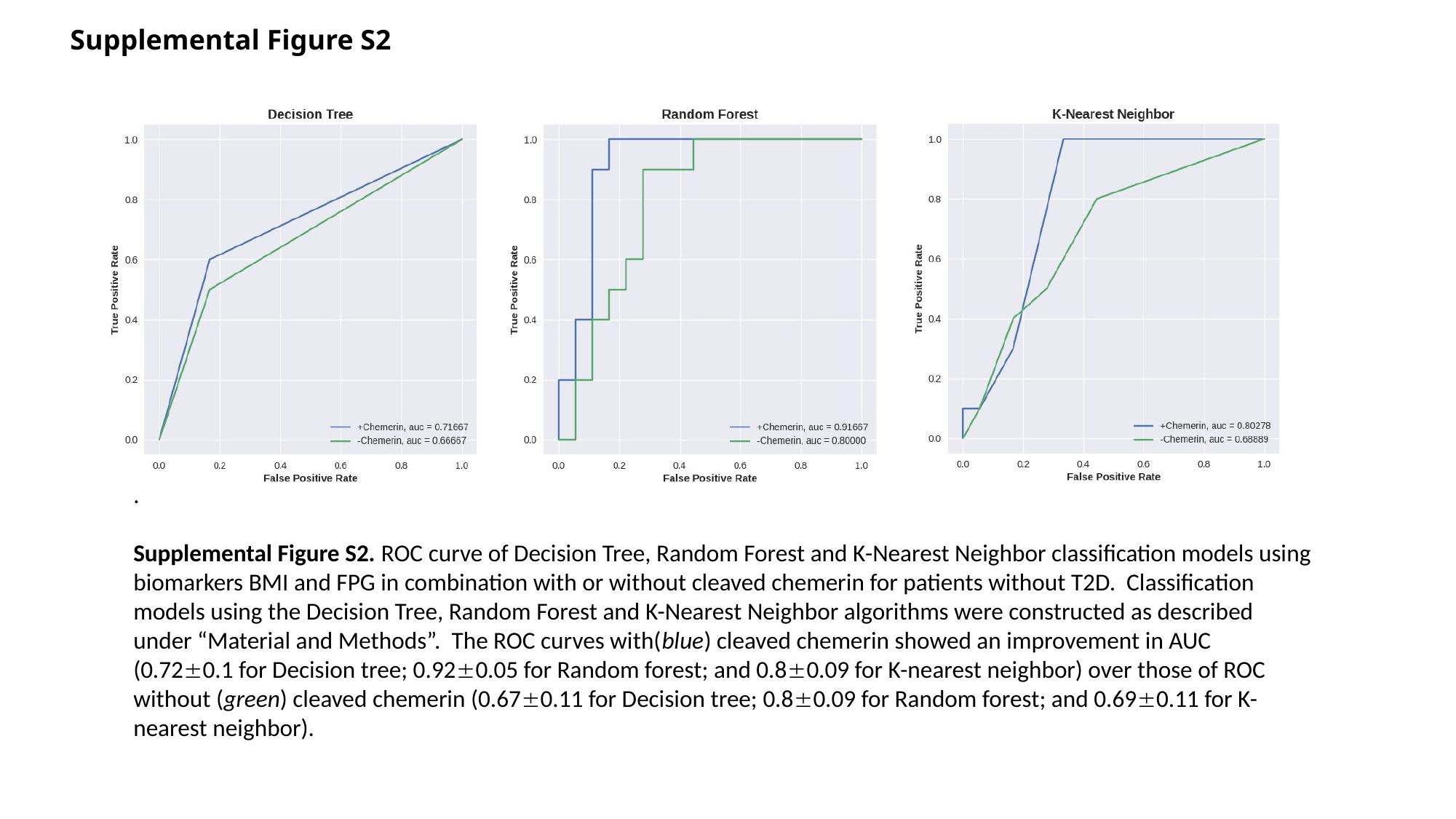

Supplemental Figure S2
.
Supplemental Figure S2. ROC curve of Decision Tree, Random Forest and K-Nearest Neighbor classification models using
biomarkers BMI and FPG in combination with or without cleaved chemerin for patients without T2D. Classification models using the Decision Tree, Random Forest and K-Nearest Neighbor algorithms were constructed as described under “Material and Methods”. The ROC curves with(blue) cleaved chemerin showed an improvement in AUC (0.72±0.1 for Decision tree; 0.92±0.05 for Random forest; and 0.8±0.09 for K-nearest neighbor) over those of ROC without (green) cleaved chemerin (0.67±0.11 for Decision tree; 0.8±0.09 for Random forest; and 0.69±0.11 for K-nearest neighbor).

## Slide 3
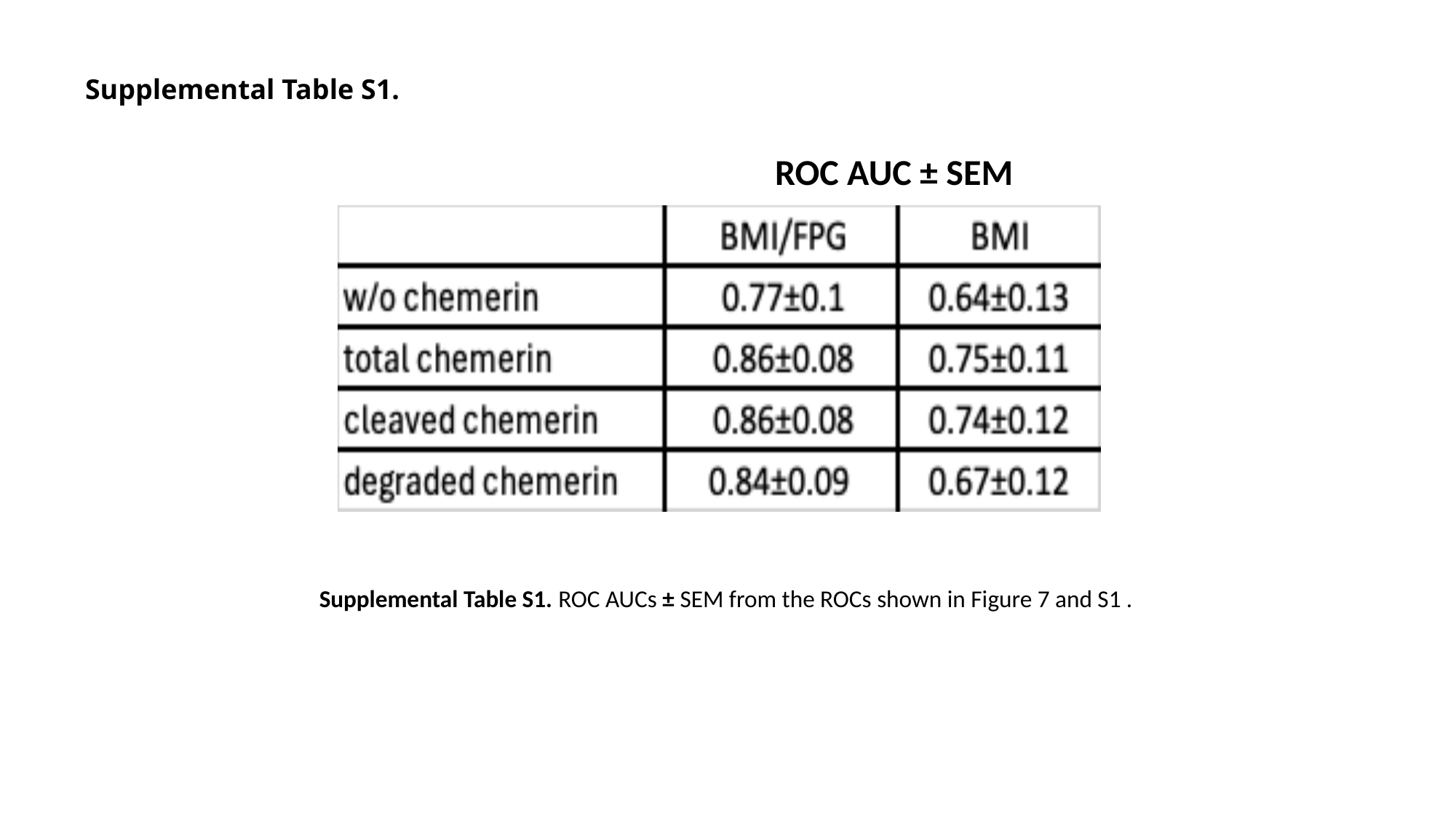

Supplemental Table S1.
ROC AUC ± SEM
Supplemental Table S1. ROC AUCs ± SEM from the ROCs shown in Figure 7 and S1 .

## Slide 4
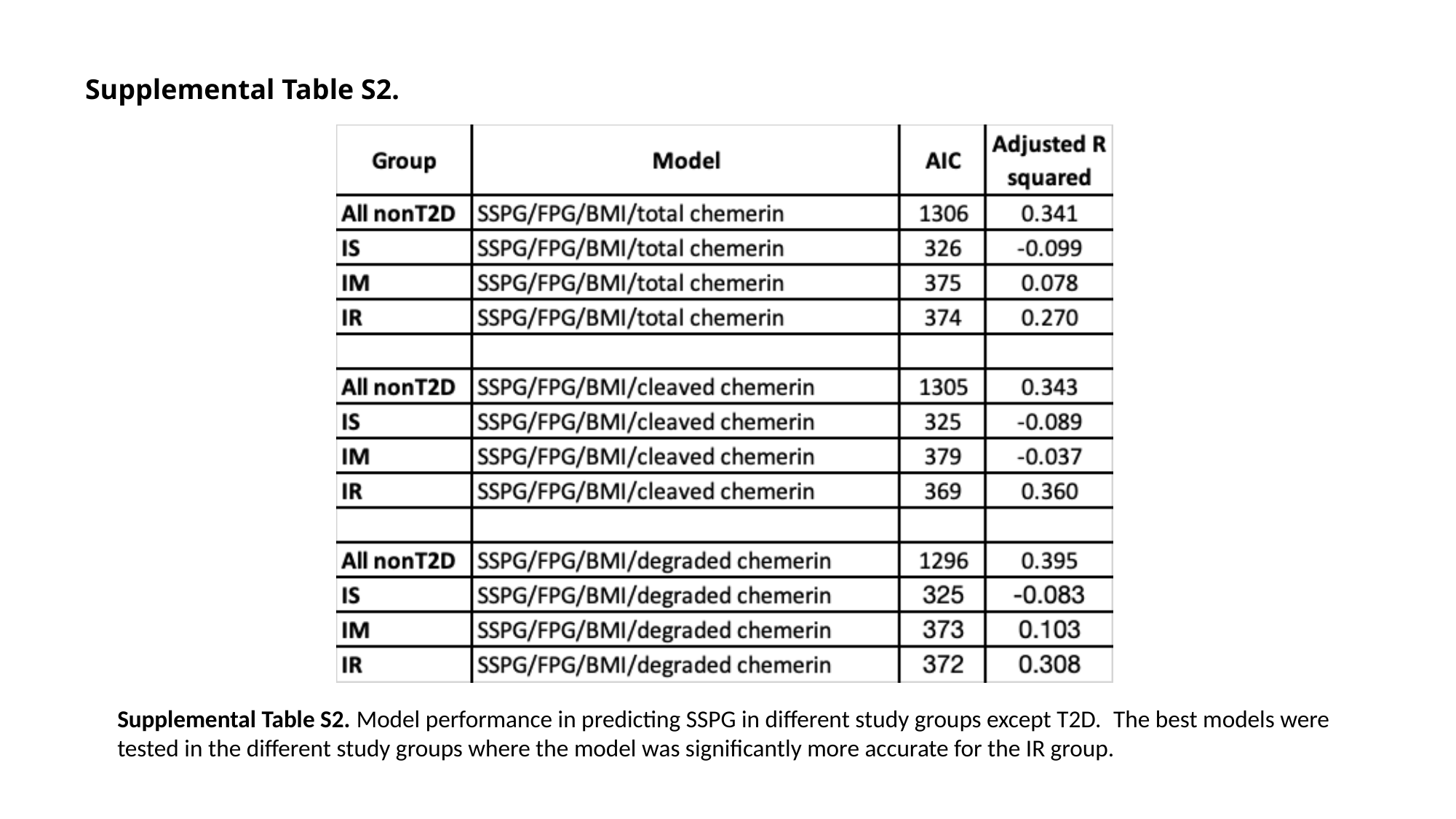

Supplemental Table S2.
Supplemental Table S2. Model performance in predicting SSPG in different study groups except T2D. The best models were tested in the different study groups where the model was significantly more accurate for the IR group.
